# Correction: Nkwinika et al. APTIMA mRNA vs. DNA-Based HPV Assays: Analytical Performance Insights from a Resource-Limited South African Setting. *Int. J. Mol. Sci.* 2025, *26*, 7450

**DOI:** 10.3390/ijms27020974

**Published:** 2026-01-19

**Authors:** Varsetile Varster Nkwinika, Kelvin Amoh Amissah, Johnny Nare Rakgole, Moshawa Calvin Khaba, Cliff Abdul Magwira, Ramokone Lisbeth Lebelo

**Affiliations:** 1 Department of Virological Pathology, Sefako Makgatho Health Sciences University, Medunsa, P.O. Box 60, Pretoria 0204, South Africa; varsetile.nkwinika@smu.ac.za (V.V.N.); 201805336@swave.smu.ac.za (K.A.A.); nare.rakgole@smu.ac.za (J.N.R.); cmagwira@gmail.com (C.A.M.); 2 South African Vaccination and Immunisation Centre (SAVIC), Sefako Makgatho Health Sciences University, Medunsa, P.O. Box 60, Pretoria 0204, South Africa; 3 Department of Anatomical Pathology, Sefako Makgatho Health Sciences University, Medunsa, P.O. Box 60, Pretoria 0204, South Africa; moshawa.khaba@nhls.ac.za; 4 National Health Laboratory Service (NHLS), Department of Anatomical Pathology, Sefako Makgatho Health Sciences University, Medunsa, P.O. Box 60, Pretoria 0204, South Africa; 5 National Health Laboratory Service (NHLS), Department of Virological Pathology, Sefako Makgatho Health Sciences University, Medunsa, P.O. Box 60, Pretoria 0204, South Africa

There was an error in the original publication [1]. The reference Sørbye et al. [21] was incorrectly described in the manuscript as using the APTIMA mRNA assay.

A correction has been made to Paragraph 7 of Section 1. Introduction:

Previous studies, including a screen-and-treat trial by Sørbye et al. [21], have demonstrated the utility of genotype-specific HPV E6/E7 mRNA testing in improving screening specificity and reducing overtreatment in South African women.

A correction has been made to Paragraph 10 of Section 3. Discussion:

This observation could obscure age-specific patterns typically observed in general populations [32,33], thus emphasizing the importance of adapting cervical cancer screening strategies, including the selection of appropriate HPV test types and screening thresholds, to local epidemiological data [21].

The authors state that the scientific conclusions are unaffected. This correction was approved by the Academic Editor. The original publication has also been updated.
